# Self-Templated Metal Glycerolate-Derived Trimetallic Layered Double Hydroxides with Tunable Metal Cation Concentration for High-Performance Supercapacitors

**DOI:** 10.1021/acsomega.6c02482

**Published:** 2026-05-16

**Authors:** Subbiramaniyan Kubendhiran, Nattha Arungwutthiwong, Thanapon Sripracha, Natkrit Kongphichphan, Hung-Ming Chen, Chutima Kongvarhodom, Yung-Fu Wu, Lu Yin Lin

**Affiliations:** † Department of Chemical Engineering and Biotechnology, 34877 National Taipei University of Technology, Taipei 10608, Taiwan; ‡ Department of Chemical Engineering, 65128 King Mongkut’s University of Technology Thonburi, 126 Pracha-u-thit, Toong-kru, Bangkok 10140, Thailand; § Gingen Technology Co., Ltd., Rm. 7, 10F., No.189, Sec. 2, Keelung Rd., Xinyi Dist., Taipei 11054, Taiwan; ∥ Department of Chemical Engineering, 56082 Ming Chi University of Technology, New Taipei City 24301, Taiwan

## Abstract

Layered double hydroxides (LDHs) are promising materials for electrochemical energy storage devices. Nonetheless, their practical application in supercapacitors is restricted by low electrical conductivity, severe aggregation, and poor internal stability. Surface modification and composition engineering are key strategies to overcome these challenges. This study presents the synthesis of flower-like trimetallic manganese nickel cobalt-layered double hydroxide (MnNiCo-LDH), derived from metal glycerolate. The NiCo-bimetal glycerolate spheres serve as both templates and metal sources for Ni and Co in the MnNiCo-LDH synthesis. These glycerolate spheres are prepared using a solvothermal method with different mole ratios (1:1, 2:1, and 1:2), and the MnNiCo-LDH is synthesized via a magnetic stirring process. The concentration of Mn was systematically varied to evaluate its effect on the structural and electrochemical properties. Under optimal conditions, MnNiCo-LDH achieves a high specific capacitance of 871.2 F/g at 20 mV/s with a broad potential window of 0.65 V. An asymmetric supercapacitor (ASC) was assembled with MnNiCo-LDH as the positive electrode and graphene as the negative electrode. The fabricated ASC demonstrates a maximum energy density of 36.9 Wh/kg at a specific power of 473.4 W/kg and exceptional cycling stability with 95.2% retention after 10,000 cycles. These results highlight the potential for developing efficient multimaterial electrodes for advanced energy storage applications.

## Graphical abstract

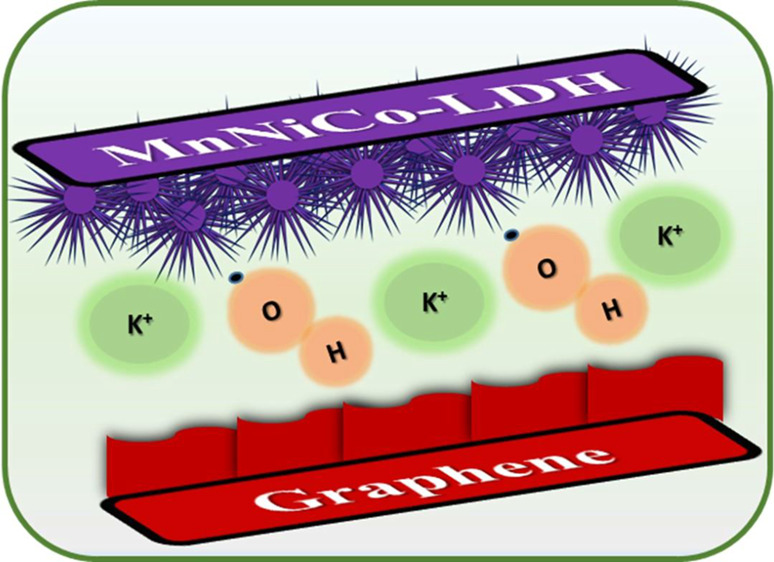

## 1 Introduction

The growing global demand for energy has highlighted the limitations of nonrenewable sources. This drives the development of advanced energy storage systems such as conventional capacitors, rechargeable batteries, and supercapacitors.^1^ Among these, supercapacitors have attracted considerable attention because of their high power density, rapid charge–discharge ability, and exceptional cycling stability.^2^ Hybrid supercapacitors, which integrate different charge storage mechanisms, typically combine electrical double-layer capacitors with pseudocapacitive materials.^3^ This design offers an effective strategy to balance high energy density with robust power performance. The electrochemical performance of such devices is largely dependent on the choice of electrode materials. In recent years, a wide range of materials, including transition metal oxides,^4^ sulfides,^5^ and layered double hydroxides (LDHs),^6^ have been extensively investigated as promising candidates for pseudocapacitor electrodes. LDHs are a kind of hydroxyl carbonate and a two-dimensional anionic layered compound resembling brucite.^7^ Their general formula is {(M^II^)_1–*x*_(M^III^)_*x*_ (OH)_2_}^*x*+^ (A^*n*–^_*x*/*n*_·*m*H_2_O), where M^II+^ and M^III+^ represent metal cations.^8–10^ Due to their excellent redox activity and substantial specific surface area, LDHs have attracted considerable interest for use in energy storage and conversion applications.^11^ In particular, adjustable chemical composition allows a modifiable ion types and interlayer structures, enhancing efficient charge-transfer pathways.^12,13^ Thus, LDHs exhibit distinguished anion exchange properties, high redox activity, and exceptional intercalation capabilities.^14^ Nevertheless, the LDHs suffer from limited electrical conductivity and structural stability, which restricts their specific capacitance.^15,16^ Moreover, volume expansion during charge–discharge cycles often leads to structural degradation, posing a challenge for their practical application in supercapacitors.^17,18^ Composition and structural engineering have emerged as effective strategies to address these limitations.^19^

Optimizing the ratio of metal cations and tailoring the interlayer anions can significantly enhance the conductivity of LDHs.^20^ This approach facilitates the formation of vacancies and the tuning of electroactive sites within the LDH structure.^6,21^ Doping active heterometallic ions into mono- or bimetallic LDHs not only expands the interlayer spacing but also alleviates mechanical stress during cycling process.^22,23^ The synergistic effect of multiple metal ions, along with efficient hydroxyl ion intercalation/deintercalation, promotes faster and more active redox processes.^24^ Additionally, the atomic ratio of metal ions strongly affects crystallinity, conductivity, and electrochemical behavior. This improves rate performance and cycling stability.^19^ Consequently, trimetallic LDHs generally outperform their mono- and bimetallic counterparts in supercapacitor applications.^25^ To date, various trimetallic LDHs have been explored for supercapacitor applications. Among them, nickel and cobalt-based hydroxides stand out due to their high theoretical capacitance and strong electrochemical activity.^26^ For example, Z. Meng et al. synthesized NiCoAl-LDH nanosheets via a one-step solvothermal method, achieving a high specific capacitance of 1001.8 F/g and 81.6% capacitance retention.^27^ Similarly, X. Wei et al. reported a high-performance hybrid supercapacitor using black phosphorus quantum dots in porous NiCoCu-LDH, where multiple redox sites enhanced capacitance.^28^ Previous studies also show that Mn incorporation improves material robustness by mitigating volume changes during cycling.^22,29^ C. Zhang et al. developed a NiCo-LDH@MnCo-LDH heterostructure that exhibited excellent structural stability and retained 93.2% of its capacitance after 20,000 cycles at 6 A/g.^30^ Based on these insights, manganese nickel cobalt-layered double hydroxide (MnNiCo-LDH) is designed as a promising cathode material for asymmetric supercapacitor (ASC). The coexistence of capacitive and battery-type charge storage mechanisms among Ni, Co, and Mn cations enhances overall performance. Additionally, valence transitions and charge hopping between these metal ions create abundant electroactive sites, thereby boosting charge storage efficiency.

Structural engineering is another key strategy for enhancing material stability, particularly by mitigating volume changes during repeated charge–discharge cycles.^30^ Designing micro and nanostructures with controlled morphologies can effectively buffer these mechanical stresses.^31^ Previously, metal–organic frameworks (MOFs) have been used as both templates and precursors for the synthesis of LDHs with porous structures.^32^ Notably, the MOF-derived LDHs retain the intricate nanostructures of their parent MOFs. Among various approaches, metal-glycerolate-derived materials have gained significant interest due to their excellent conductivity, electrochemical activity, and structural stability.^33^ These metal-glycerolates act as sacrificial templates, enabling the synthesis of diverse micro/nanomaterials with tailored architectures.^34,35^ For example, C. Cheng et al.^34^ developed Ni–Mn hydroxide hollow spheres from metal glycerolates for supercapacitor applications. Similarly, L. Shen et al.^36^ reported NiCo_2_S_4_ ball-in-ball hollow spheres, while L. Hou et al.^37^ fabricated asymmetric supercapacitors using flower-like NiCo-LDH structures. On the other hand, Tong Li et al. synthesized a p-n heterojunction by integrating Mn-glycerolate-derived MnS microspheres with Ni-MOF for supercapacitor applications.^38^ The unique design promotes charge redistribution at the p–n junction, forming a space-charge region and built-in electric field that enhance charge transfer and improve intrinsic activity. These hollow and flower-like morphologies enhance electrochemical performance by improving electrolyte accessibility and accommodating volume fluctuations during cycling. To address the limitations of conventional LDH electrodes, this work proposes a metal glycerolate-derived strategy to construct a flower-like trimetallic MnNiCo-LDH. The study systematically investigates the role of Mn incorporation in modulating the structural, morphological, and electrochemical properties of the LDH. By optimizing the Ni:Co ratio and Mn content, the work aims to enhance charge transport, increase the number of active sites, and improve structural stability. Furthermore, the practical applicability of the optimized material is evaluated by fabricating an ASC device. The device aims to deliver high energy density, good rate capability, and long-term cycling stability. This approach provides insights into the design of advanced multimetallic LDH systems for next-generation energy storage applications.

## 2 Experimental Section

The list of chemical reagents used for the synthesis of MnNiCo-LDH and the characterization techniques used in this were provided in the Supporting Information (SI).

### 2.1 Synthesis of Nickel–Cobalt Glycerolate (NiCo-Glycerolate) Spheres

In a typical procedure, an equal mole (0.5 mmol) ratio (1:1) concentration of Ni­(NO_3_)_2_·6H_2_O, and Co­(NO_3_)_2_·6H_2_O was dissolved in 52.5 mL of isopropyl alcohol. Next, a specific amount of glycerol was slowly added to the above solution, which was stirred vigorously using a magnetic stirrer to achieve a homogeneous solution. Following this, the homogeneous solution was placed in a Teflon-lined stainless-steel autoclave, and the solvothermal process was conducted at 180 °C for 7 h. The mixture was cooled to room temperature, then the product was collected, washed twice with water and ethanol, and dried at 60 °C for 12 h. Under similar conditions, the NiCo-glycerolate spheres were prepared by varying the Ni to Co mole ratios, such as 1:2 and 2:1.

### 2.2 Preparation of Hierarchical Flower-like MnNiCo LDH

In this work, highly uniform NiCo-glycerolate spheres were used as sacrificial templates for the synthesis of MnNiCo-LDH. In a typical procedure, 50 mg of NiCo-glycerolate (1:1) spheres were dispersed in 50 mL of 0.01 M Mn­(NO_3_)_2_·4H_2_O and mildly stirred for 6 h at ambient condition. After 6 h, the MnNiCo-LDH product was separated and washed twice with absolute ethanol. The washed product was dried in an oven at 60 °C for 5 h. The synthesized MnNiCo-LDH is denoted as MnNiCo-LDH-A. Similarly, NiCo-glycerolate (1:2) and NiCo-glycerolate (2:1) were used for the synthesis of MnNiCo-LDH denoted as MnNiCo-LDH-B and MnNiCo-LDH-C, respectively. Further, the MnNiCo-LDH was prepared with different concentrations of Mn­(NO_3_)_2_·4H_2_O solution, including 0.005 and 0.015 M. The MnNiCo-LDH was prepared using NiCo-glycerolate (1:1) and 0.005 M Mn­(NO_3_)_2_·4H_2_O, denoted as MnNiCo-LDH-D. In addition, MnNiCo-LDH-E was synthesized using 0.015 M (Mn­(NO_3_)_2_·4H_2_O. The synthesis process and application of MnNiCo-LDH are illustrated in Figure 1. The specific concentrations of Ni, Co, and Mn precursors used for the synthesis of MnNiCo-LDHs are summarized in Table 1.

**Figure 1:**
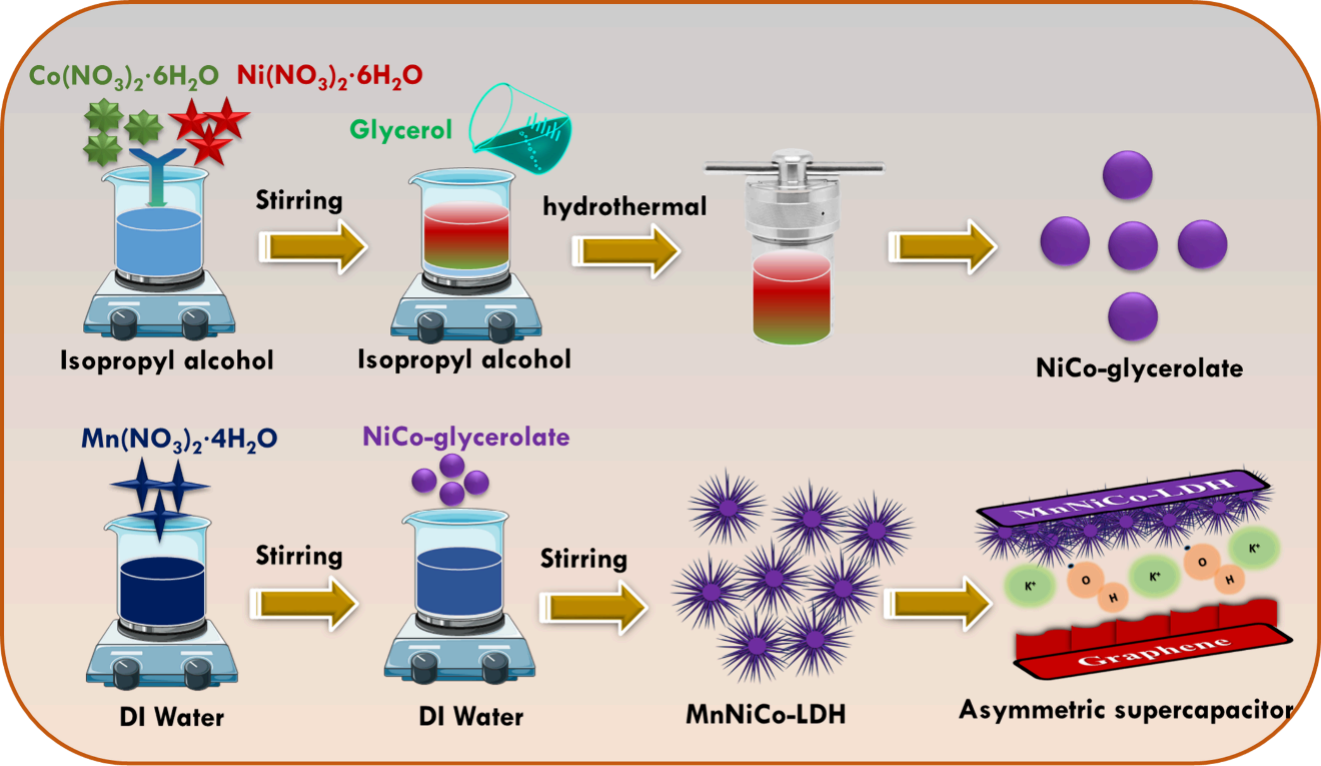
Schematic illustration of MnNiCo-LDH synthesis and application.

**Table 1:** Metal Precursor Concentrations Used for Synthesizing Trimetallic MnNiCo-LDH Materials

**material name**	**Ni(NO**_**3**_ **)**_**2**_ **·6H**_**2**_ **O** **(mmole)**	**Co(NO**_**3**_ **)**_**2**_ **·6H**_**2**_ **O** **(mmole)**	**Mn(NO**_**3**_ **)**_**2**_ **·4H**_**2**_ **O** **(M)**
MnNiCo-LDH-A	0.50	0.50	0.010
MnNiCo-LDH-B	0.25	0.50	0.010
MnNiCo-LDH-C	0.50	0.25	0.010
MnNiCo-LDH-D	0.50	0.50	0.005
MnNiCo-LDH-E	0.50	0.50	0.015

## 3 Results and Discussion

### 3.1 Structure and Morphology Analysis

The surface morphology of the synthesized materials was examined using FESEM, and the results are shown in Figure 2. As observed in Figure 2a, NiCo-glycerolate (1:1) exhibits uniform-sized spherical structures. When Ni and Co precursors are dissolved in isopropyl alcohol, Ni^2+^ and Co^2+^ form metal isopropoxides via exchange reactions. These species then undergo ligand exchange with glycerol due to its higher boiling point, resulting in NiCo-glycerolate formation. These hydrothermally synthesized NiCo-glycerolate spheres were then used to prepare flower-like MnNiCo-LDH. As shown in Figure 2, MnNiCo-LDH-A, MnNiCo-LDH-B, MnNiCo-LDH-C, MnNiCo-LDH-D, and MnNiCo-LDH-E samples exhibit consistent flower-like morphology. While stirring, hydroxide ions (OH^–^) from water facilitate the hydrolysis of glycerolate. Over time, Mn^2+^ ions react with the released Ni^2+^/Ni­(OH)_2_ and Co^2+^/Co­(OH)_2_, forming nanosheets on the spheres and producing flower-like structures. Notably, variations in the concentrations of NiCo-glycerolate or Mn­(NO_3_)_2_·4H_2_O did not significantly alter the resulting morphology. To further investigate the surface morphology, elemental distribution, and composition, TEM analysis was performed, and the corresponding results are presented in Figure 3. As shown in Figure 3a–c, the core spheres are decorated with nanosheets, forming a distinct flower-like structure of MnNiCo-LDH. The HRTEM image (Figure 3d) shows distinct lattice fringes of MnNiCo-LDH. The measured interplanar spacings of 0.258 and 0.227 nm correspond to the (012) and (015) planes, respectively. These results are consistent with the XRD pattern. Figure 3e–h illustrates the elemental mapping results of Ni, Co, Mn, and O in the MnNiCo-LDH confirming the even distribution of these elements throughout the structure. Additionally, the elemental composition was determined from the EDX spectrum (Figure 3i), which revealed weight percentages of 23.4%, 19.5%, 11.3%, and 45.8% for Ni, Co, Mn, and O elements, respectively. These TEM and EDX results confirm the successful formation of the flower-like MnNiCo-LDH structure with homogeneous elemental distribution.

**Figure 2:**
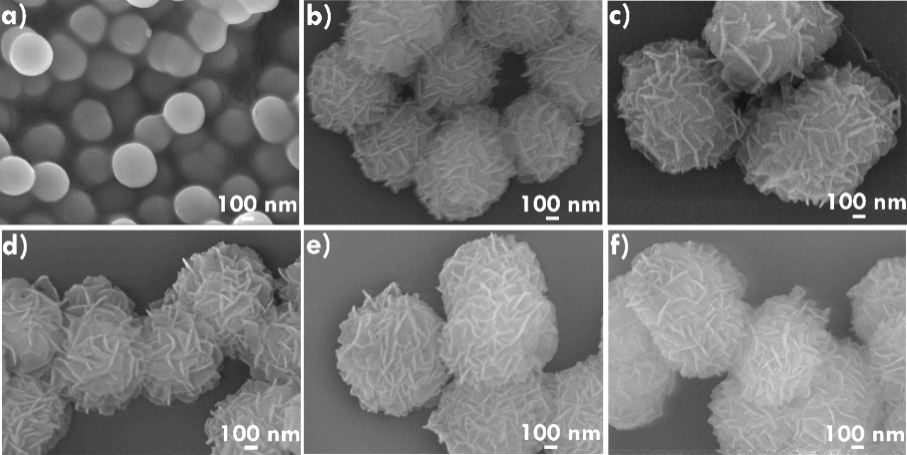
FESEM images of (a) NiCo-glycerolate, (b) MnNiCo-LDH-A, (c) MnNiCo-LDH-B, (d) MnNiCo-LDH-C, (e) MnNiCo-LDH-D, and (f) MnNiCo-LDH-E.

**Figure 3:**
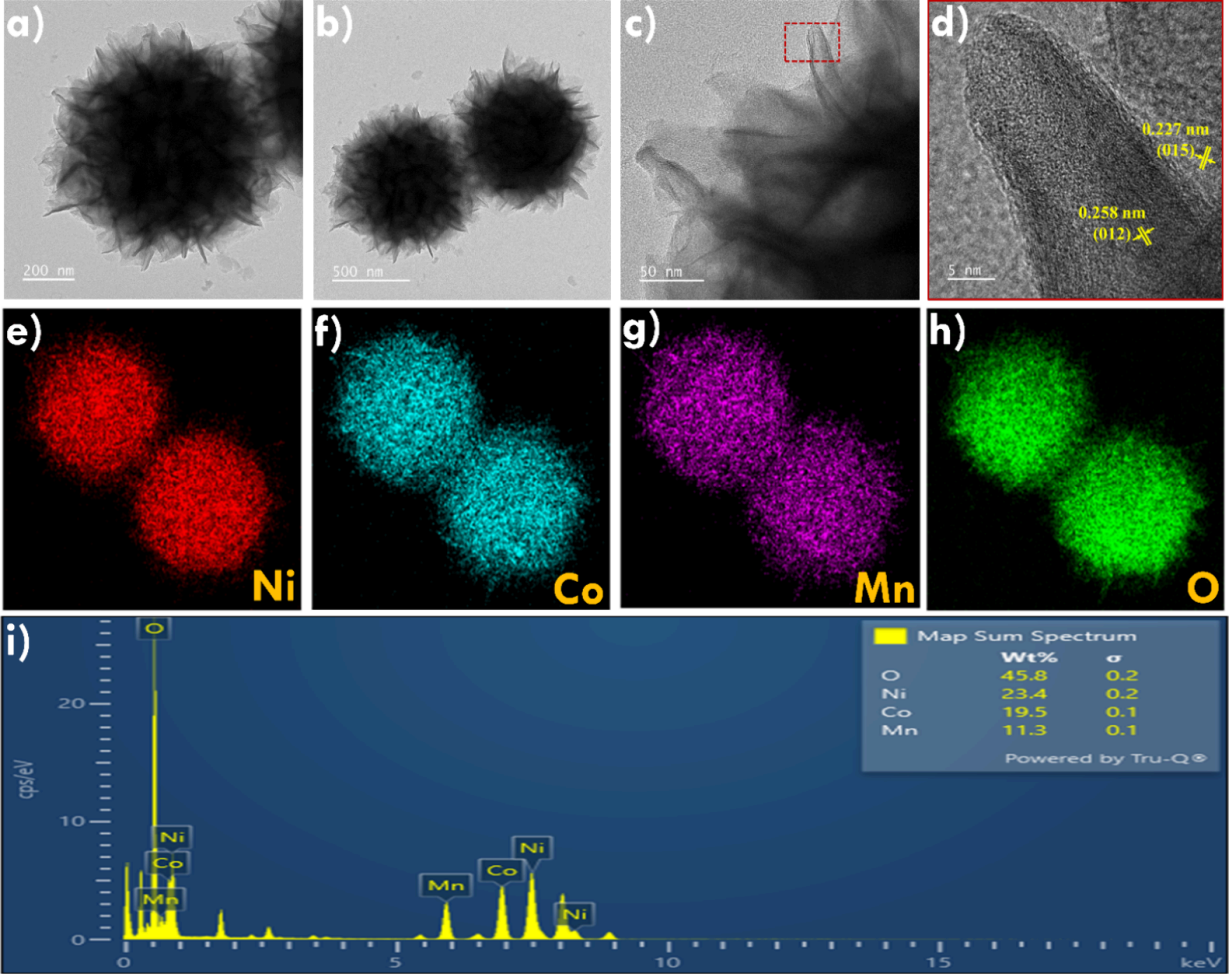
(a–c) TEM images of MnNiCo-LDH-A recorded at higher and lower magnification; (d) HRTEM image of MnNiCo-LDH-A; EDX mapping results of (e) Ni, (f) Co, (g) Mn, and (h) O elements presented in MnNiCo-LDH-A; (i) EDX elemental spectrum of MnNiCo-LDH-A.

The flower-like structures formed after the mild stirring process for 6 h in the presence of Mn­(NO_3_)_2_·4H_2_O solution. During the stirring process, the NiCo-glycerolate spheres react with Mn­(NO_3_)_2_·4H_2_O and produced trimetallic MnNiCo-LDH. X-ray diffraction (XRD) analysis was conducted to check the synthesized materials’ phase composition and crystal structure. Figure 4a shows the XRD pattern of MnNiCo-LDH synthesized using different Ni and Co mole ratios. The diffraction peaks of MnNiCo-LDH-A were found at 11.37°, 23.36°, 34.25°, 38.88°, 45.93°, and 60.37°, which can be associated respectively to the (003), (006), (012), (015), (018), and (110) planes of hydrotalcite-like LDH. Earlier investigations have reported that binary and ternary LDHs display comparable diffraction patterns.^39,40^ Thus, the peaks were indexed based on the standard NiCo-LDH pattern (JCPDS No. 40-0216).^39,41^ A similar pattern was observed for the MnNiCo-LDH-B and MnNiCo-LDH-C. However, the peak at 11.37° shifted to 11.54° and 11.68° in the XRD patterns of MnNiCo-LDH-B and MnNiCo-LDH-C, respectively, due to the changes in the interlayer distance. These results confirm that varying the Ni and Co mole ratio leads to a change in the interlayer distance of MnNiCo-LDH. Figure 4b shows the XRD pattern of MnNiCo-LDH synthesized using different molar concentrations of Mn. Compared with MnNiCo-LDH-A, the lower (0.005 M) and higher (0.015 M) Mn concentrations lead to the peak shifts at 11.58° and 11.65°, respectively. Changes in the ionic radius and M–OH bonding can cause changes in the interlayer distance, leading to the peak shifts to higher theta values in the XRD pattern.^26^ To confirm the conversion of NiCo-glycerolate to MnNiCo-LDH, XRD and Fourier-transform infrared (FTIR) spectroscopy analyses were conducted. As shown in Figure S1, the XRD pattern of NiCo-glycerolate does not exhibit any distinct diffraction peaks, indicating its amorphous nature, which is consistent with previous reports.^42^ Following stirring of NiCo-glycerolate in Mn­(NO_3_)_2_·4H_2_O solution, the product showed well-defined hydrotalcite-like LDH peaks, confirming the formation of MnNiCo-LDH (Figure S1a). The FTIR spectrum of NiCo-glycerolate shows multiple absorption bands at various wavenumbers (Figure S1b). A broad peak at 3401 cm^–1^ corresponds to O–H stretching vibrations, while a peak at 2861 cm^–1^ is assigned to C–H stretching. Bands in the range of 1644–1585 cm^–1^ are attributed to C=O and C=C stretching vibrations. Additional peaks observed in the regions of 1288–1450, 1048–1118, and 804–1012 cm^–1^ correspond to C–H bending, C–O stretching, and C–C stretching vibrations, respectively.^43^ New peaks at 1604 and 1351 cm^–1^ are assigned to H–O–bH bending and nitrate (NO_3_^–^) vibrations in MnNiCo-LDH-A. The FTIR spectrum also shows an O–H stretching band at 3486 cm^–1^, confirming the incorporation of hydroxide. The disappearance of C=O, C=C, and nitrate bands indicates the conversion of NiCo-glycerolate to MnNiCo-LDH. X-ray photoelectron spectroscopy (XPS) technique was employed to analyze the surface elemental composition and their corresponding oxidation states of MnNiCo-LDH. Figure S2 in the SI shows the survey spectrum of MnNiCo-LDH-A, where the peaks for Co, Mn, Ni, and O confirm their presence in the synthesized MnNiCo-LDH. Figure 4c shows the high-resolution Co 2p XPS spectrum showing the peaks at 780.12 and 796 corresponding to Co 2p_3/2_ and Co 2p_1/2_, respectively. The peaks at 779.8 7 and 795.5 eV represent the Co^3+^, while those at 782.37 and 797.35 eV are allocated to the Co^2+^.^44^ The satellite peaks of Co 2p appeared at the binding energies of 786.37 and 801.12 eV. As shown in Figure 4d, the Ni 2p spectrum shows the peaks at 854.62 and 872.7 eV attributed to Ni 2p_3/2_ and Ni 2p_1/2_, respectively. The fitting peaks of the Ni^2+^ peaks appeared at 854. 37 and 872.5 eV, while the Ni^3+^ peaks at 856.3 and 874.12 eV.^45^ Further, Ni 2p shows the satellite peaks at 860.62 and 879.62 eV. Figure 4e demonstrates the core-level XPS spectrum of Mn 2p, where the Mn 2p_3/2_ and Mn 2p_1/2_ are observed respectively at 641.25 and 652.62 eV. In Mn 2p_3/2_, the peaks at 640.87, 643.37, and 646.5 eV were assigned respectively for Mn^2+^, Mn^3+^, and Mn^4+^.^46^ The deconvoluted O 1s spectrum exhibits three peaks at binding energies of 530.37, 531.62, and 532.37 eV, corresponding to O_a_ (metal oxygen), O_b_ (metal hydroxide), and O_c_ (surface water) molecules, respectively (Figure 4f).^47^ The higher intensity of the hydroxide peak O_b_ in the O 1s spectrum confirms the hydroxide-rich layered structure of the LDH. Moreover, the abundant −OH groups enhance electrolyte wettability and provide active sites for redox reactions, thereby improving electrochemical performance. The XPS results confirm the existence of the elements and their corresponding oxidation states.

**Figure 4:**
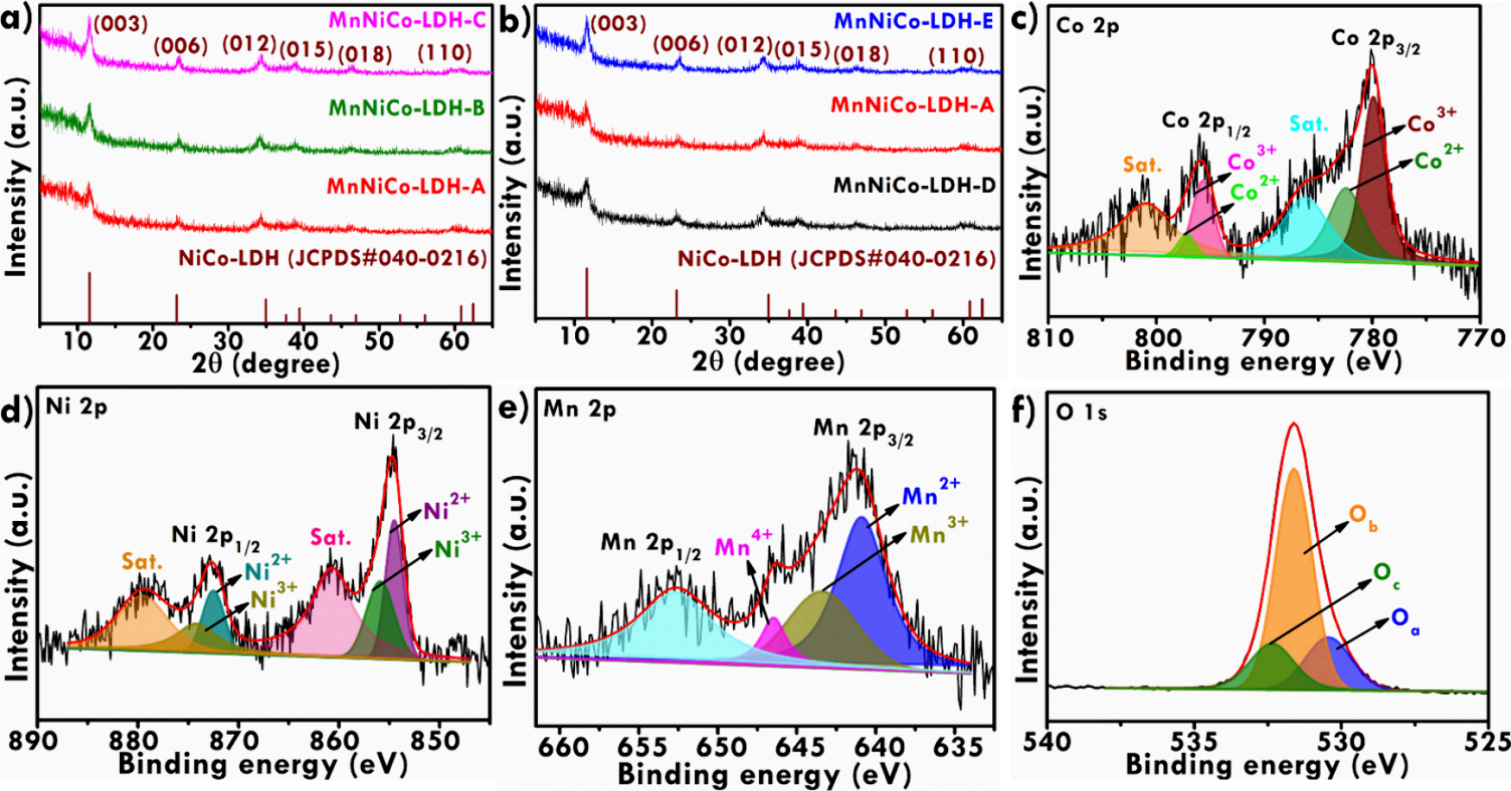
XRD patterns of (a) MnNiCo-LDH synthesized using different mole concentrations of Ni and Co precursor; XRD patterns of (b) MnNiCo-LDH synthesized using different molar concentrations of Mn precursor; high-resolution XPS spectrum of (c) Co 2p, (d) Ni 2p, (e) Mn 2p, and (f) O 1s existed in MnNiCo-LDH-A.

### 3.2 Electrochemical Analysis

The capacitance of the synthesized materials was evaluated by CV and GCD techniques in a 3 M KOH solution using a three-electrode electrochemical system arrangement. Figure 5a displays the CV curves of MnNiCo-LDH-A, MnNiCo-LDH-B, MnNiCo-LDH-C, MnNiCo-LDH-D, and MnNiCo-LDH-E electrodes recorded at 20 mV/s. All the electrodes exhibit redox peaks that conform to their pseudocapacitive behavior, resulting from the redox reactions of the Co (Co^2+^/Co^3+^), Ni (Ni^2+^/Ni^3+^), and Mn (Mn^2+^/Mn^3+^/Mn^4+^) ions. These metals present in the MnNiCo-LDH react with the alkaline electrolyte and produce M–OH and M–OOH (where M corresponds to the Co, Ni, and Mn). The electrochemical redox process of the electroactive elements is demonstrated in eqs 1 to 4 as follows.^48,49^

$$\mathrm{Co}{\left(\mathrm{OH}\right)}_{2}+{\mathrm{OH}}^{-}↔\mathrm{CoOOH}+H_{2}O+e^{-}$$ 1

$$\mathrm{Ni}{\left(\mathrm{OH}\right)}_{2}+{\mathrm{OH}}^{-}↔\mathrm{NiOOH}+H_{2}O+e^{-}$$ 2

$$\mathrm{Mn}{\left(\mathrm{OH}\right)}_{2}+{\mathrm{OH}}^{-}↔\mathrm{MnOOH}+H_{2}O+e^{-}$$ 3

$$\mathrm{MnOOH}+{\mathrm{OH}}^{-}↔{\mathrm{MnO}}_{2}+H_{2}O+e^{-}$$ 4

**Figure 5:**
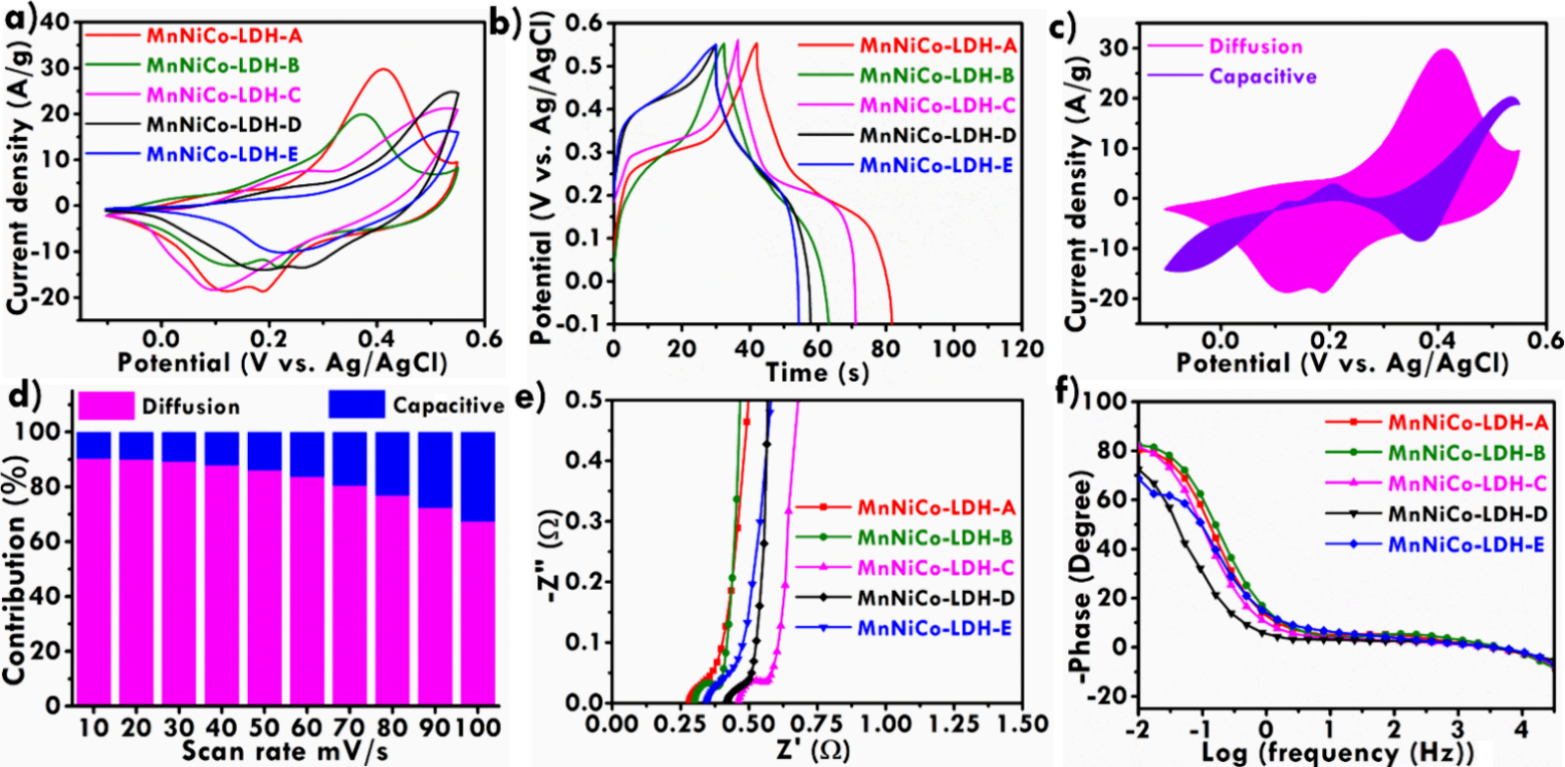
(a) CV curves recorded at 20 mV/s and (b) GCD curves recorded at 7.5 A/g of MnNiCo-LDH; (c) diffusion and capacitive contribution ratio recoded at 20 mV/s and (d) relationship between the contribution ratio and scan rates of MnNiCo-LDH-A; (e) Nyquist plots and (f) Bode plots of MnNiCo-LDH.

As can be seen, the CV integral area changed with changes in the Ni and Co molar ratios. In comparison, the MnNiCo-LDH-A shows a higher integral area than the MnNiCo-LDH-B and MnNiCo-LDH-C. On the other hand, the integral area of the MnNiCo-LDH-D and MnNiCo-E decreased when compared with MnNiCo-LDH-A due to the changes in the molar concentration of Mn. Based on the CV integral area, the specific capacitance (*C*_F_) of the electrodes was calculated using the following eq 5.^50^

$$C_{F}=\frac{\int IdV}{\Delta V\cdot \nu }$$ 5

In this equation, ν represents the scan rate, ∫*I*d*V* denotes the integrated area, and Δ*V* signifies the potential window. The calculated *C*_F_ values of the MnNiCo-LDH-A, MnNiCo-LDH-B, MnNiCo-LDH-C, MnNiCo-LDH-D, and MnNiCo-LDH-E electrodes are 871.2, 618.2, 555.1, 574.6, and 370.8 F/g, respectively. The electrochemical performance of MnNiCo-LDH electrodes is strongly influenced by the redox activity and synergistic interactions of the transition metal ions Ni, Co, and Mn. Each of these metals exhibits multiple valence states, Ni^2+^/Ni^3+^, Co^2+^/Co^3+^, and Mn^2+/^Mn^3+^/Mn^4+^, which contribute to Faradaic charge storage during the electrochemical process. These coupled redox reactions enhance specific capacitance and enable a wider potential window of 0.65 V in this study. The flower-like MnNiCo-LDH offers abundant active sites and an improved layered framework, enabling smooth and reversible ion intercalation/deintercalation. These structural features directly contribute to enhanced charge storage and transport dynamics. To investigate the effect of Ni:Co molar ratios, MnNiCo-LDHs were synthesized using three different precursor ratios. Among them, the sample MnNiCo-LDH-A, synthesized with a 1:1 mol ratio of Ni and Co, demonstrated the highest specific capacitance. This improved performance can be attributed to the optimal balance between Ni and Co redox centers, which both actively participate in the charge storage mechanism. The equal mole ratio improves crystallinity and LDH ordering, facilitating ion diffusion and efficient charge transport. This structural regularity likely enhances the electronic conductivity and overall electrochemical activity of the materials. In contrast, when the Ni:Co ratio deviates from 1:1, the layered structure of the LDHs is disrupted, leading to lower specific capacitance. Notably, Co exhibits highly reversible redox behavior and enhances electrical conductivity. Accordingly, MnNiCo-LDH-B (Co-rich) demonstrates superior performance compared to MnNiCo-LDH-C (Ni-rich), likely due to improved electron mobility and accelerated redox kinetics. To probe Mn content effects, MnNiCo-LDH-D and MnNiCo-LDH-E were synthesized using 0.005 and 0.015 M Mn precursor, respectively, with a fixed Ni:Co ratio of 1:1. The MnNiCo-LDH-A sample, with 0.01 mM Mn, outperformed both. The lower performance of MnNiCo-D (0.005 M Mn) could be due to insufficient Mn incorporation, leading to fewer active redox sites and weaker structural modification of the LDH. Conversely, the lower performance of MnNiCo-LDH-E (0.015 M Mn) likely arises from excess Mn-induced defects that disrupt the crystal structure and hinder conductivity. High Mn loading may also block the active sites or hinder ion diffusion pathways, resulting in poor utilization of the electroactive material. Therefore, the optimized composition with a Ni:Co molar ratio of 1:1 and a Mn precursor concentration of 0.01 M (MnNiCo-LDH-A) demonstrates superior electrochemical behavior. Synergistic redox species, an ordered LDH structure, and stable flower-like morphology enhance capacitance. This highlights the importance of compositional and structural tuning for high-performance LDH electrodes. Figure 5b illustrates the GCD curves of MnNiCo-LDH-A, MnNiCo-LDH-B, MnNiCo-LDH-C, MnNiCo-LDH-D, and MnNiCo-LDH-E electrodes at a current density of 7.5 A/g. At low current densities, reaching the upper potential of the wide potential window is challenging. Therefore, a low current density of 7.5 A/g was used for GCD analysis. The capacitance values are calculated from the GCD results using the eq 6.^50^

$$C_{F}=\frac{I\cdot t}{\Delta V}$$ 6

As shown in Figure 5b, two pairs of plateaus were observed in all GCD curves, which align with the peaks in the CV curves. In addition, the IR drop values 0.03, 0.05, 0.06, 0.08, and 0.09 V were measured from GCD curves of MnNiCo-LDH-A, MnNiCo-LDH-B, MnNiCo-LDH-C, MnNiCo-LDH-D, and MnNiCo-LDH-E, respectively. Among all, the MnNiCo-LDH-A has the lowest IR drop due to its low internal resistance. Notably, the MnNiCo-LDH-A electrode exhibits the longest discharge time of the other electrodes. The driving force is inadequate at a low applied current density, therefore, it is very hard for the electrode to reach a high potential. On the other hand, at a high applied current density, the rapid charging and discharging process can negatively affect the energy storage capacity. Despite the higher current density used in GCD, the electrodes exhibited appreciable specific capacitance. Values of 459.5, 354.2, 347.3, 321.9, and 280.4 F/g were obtained for MnNiCo-LDH-A, MnNiCo-LDH-B, MnNiCo-LDH-C, MnNiCo-LDH-D, and MnNiCo-LDH-E, respectively. To evaluate the contribution ratio from the capacitive and diffusion-controlled processes on the MnNiCo-LDH-A electrode, Dunn’s method was used as follows (eq 7).^51^

$$I(V)=k_{1}\nu +k_{2}\nu^{0.5}$$ 7

In this context, *I* denote the current density as the variable. Here, *k*_1_ν and *k*_2_ν^0.5^ represent the influences of surface capacitive behavior and diffusion-controlled processes, respectively. Figure 5c illustrates the CV curves demonstrating the capacitive and diffusion ratio at the scan rate of 20 mV/s. It was around 90.29% of the total charge that was stored via diffusion, whereas 9.71% arose from capacitive processes. The relative contribution ratios at various scan rates were assessed, and the findings are presented in Figure 5d. It is evident that as the scan rate rises, the diffusion-controlled contribution diminishes. This trend occurs because a faster scan rate reduces the time for redox reactions, leading to fewer available oxidation and reduction sites. The electron transport property of the electrodes is related to the electrochemical performance of the electrodes in the supercapacitor application. Therefore, the charge transport of the electrodes was investigated by electrochemical impedance spectroscopy (EIS) analysis. Figure 5e depicts the Nyquist plots of MnNiCo-LDH-A, MnNiCo-LDH-B, MnNiCo-LDH-C, MnNiCo-LDH-D, and MnNiCo-LDH-E electrodes, while the inset shows the Randles equivalent circuit model. In the equivalent circuit model, the CPE, *R*_ct_, *R*_s_, and *Z*_w_ represent the constant phase element, charge transfer resistance, solution resistance, and Warburg diffusion processes, respectively.^52^ The *R*_s_ value is measured from the intercept of the Nyquist plot at low frequency. From the Nyquist plots, the *R*_s_ values 0.27, 0.29, 0.45, 0.47, and 0.34 Ω were measured for MnNiCo-LDH-A, MnNiCo-LDH-B, MnNiCo-LDH-C, MnNiCo-LDH-D, and MnNiCo-LDH-E electrodes, respectively. The *R*_ct_ of the electrode is equal to the semicircle portion of the electrode. Notably, the *R*_ct_ values increase when the semicircle portion of the Nyquist plot increases. The *R*_ct_ values of 0.11, 0.41, 0.6, 0.48, and 0.55 Ω were calculated for MnNiCo-LDH-A, MnNiCo-LDH-B, MnNiCo-LDH-C, MnNiCo-LDH-D, and MnNiCo-LDH-E, respectively. Owing to the equal mole ratio of Ni and Co, an optimized concentration of Mn improved the conducting property of the MnNiCo-LDH-A. Since the Co increases the electrical conductivity, the MnNiCo-LDH-B shows a lower *R*_ct_ than the MnNiCo-LDH-C, which contains a high concentration of Ni. When varying the Mn concentration, the low concentration (MnNiCo-LDH-D) exhibited a lower *R*_ct_ value than the higher Mn concentration (MnNiCo-LDH-E). The higher concentration of Mn could suppress the active sites and disrupt the conductive pathways of Ni–Co LDH. Therefore, the *R*_ct_ value of the MnNiCo-LDH increased at higher concentrations of Mn. The low IR drop indicates reduced internal resistance. This is due to improved conductivity and better electron transport from the synergistic interaction of Ni, Co, and Mn. The small *R*_ct_ value suggests faster charge transfer at the electrode–electrolyte interface. This is attributed to multiple redox-active sites in the trimetallic system. These factors lead to enhanced electrochemical performance, including higher capacitance and improved rate capability. Therefore, MnNiCo-LDH-A exhibited an enhanced supercapacitor performance. The specific capacitance, capacity, *R*_s_ and *R*_ct_ values of the fabricated electrodes are compared in Table 2. Figure 5e shows the Bode phase angle plots of the MnNiCo-LDH-A, MnNiCo-LDH-B, MnNiCo-LDH-C, MnNiCo-LDH-D, and MnNiCo-LDH-E electrodes. Among them, MnNiCo-LDH, MnNiCo-LDH, and MnNiCo-LDH show the values around 80°, while the MnNiCo-LDH-D and MnNiCo-LDH-E electrode phase angle values are 70°. It can be seen that the phase angle values of the electrodes are close to 90°, revealing the excellent capacitive behavior of the MnNiCo-LDH electrodes.^53^

**Table 2:** Electrochemical Performance Comparison of the CoNiMn-LDH Synthesized at Different Co, Ni, and Mn Concentrations

**active material**	**loading mass (mg)**	***C***_**F**_ **(F/g)** ^a^	**capacity** (mAh/g)^a^	***C***_**F**_ **(F/g)** ^b^	**capacity** (mAh/g)^b^	***R***_**s**_ **(Ω)**	***R***_**ct**_ **(Ω)**
CoNiMo-LDH-A	6.4	871.2	157.3	459.5	83.0	0.27	0.11
CoNiMo-LDH-B	10.0	618.2	111.6	354.2	64.0	0.29	0.41
CoNiMo-LDH-C	8.6	555.1	100.2	347.3	62.7	0.45	0.60
CoNiMo-LDH-D	8.4	574.6	103.7	321.9	58.1	0.47	0.48
CoNiMo-LDH-E	8.4	370.8	67.0	280.4	50.6	0.34	0.55

a Calculated at 20 mV/s.

b Calculated at 7.5 A/g.

The rate performance of the MnNiCo-LDH electrodes was assessed by CV and GCD techniques. Figure S3a–e respectively shows the CV curves of MnNiCo-LDH-A, MnNiCo-LDH-B, MnNiCo-LDH-C, MnNiCo-LDH-D, and MnNiCo-LDH-E electrodes recorded at scan rates ranging from 10 to 100 mV/s. As can be seen, the current density of the MnNiCo-LDH electrodes increases with increasing scan rates, indicating the good reversibility and fast redox kinetics of the materials. Notably, at lower scan rates (10 to 50 mV/s), significant changes are evident in both the current density and the integrated area under the CV curves. This reflects enhanced faradaic contributions, as slower scan rates allow more time for the electroactive species to participate in redox reactions and for efficient ion diffusion. However, at higher scan rates, these changes become subtle. This is due to reduced ion-electrode interaction time, which suppresses Faradaic reactions and favors surface capacitive behavior.^54^ At high scan rates, the linearly increasing capacitive current dominates, overshadowing the faradaic contribution. This phenomenon may result in the CV curves appearing less responsive to further increases in scan rate. Moreover, fast scan rates restrict the diffusion of ions to the inner active sites, thereby reducing the overall charge storage efficiency. Figure S3f illustrates the variation in specific capacitance across different scan rates derived from the CV measurements. The specific capacitance retention values are calculated to be 29.7%, 26.6%, 18.6%, 22.5%, and 31.7% for MnNiCo-LDH-A, MnNiCo-LDH-B, MnNiCo-LDH-C, MnNiCo-LDH-D, and MnNiCo-LDH-E, respectively. Among these, MnNiCo-LDH-A exhibited the highest capacitance retention, indicating optimal charge-transfer capability and structural integrity under fast redox cycling. When the Ni:Co ratio deviates from 1:1, the structural balance and electronic conductivity are disrupted, leading to lower capacitance retention. However, higher Co content in MnNiCo-LDH-B enhances electrical conductivity and accelerates charge-transfer kinetics, resulting in better capacity retention compared to MnNiCo-LDH-C. This supports the known role of Co in enhancing rate performance. Similarly, MnNiCo-LDH-E exhibits higher capacitance retention than MnNiCo-LDH-D. This could be attributed to the increased presence of Mn in MnNiCo-LDH-E, which may improve structural stability. Nevertheless, at lower scan rates, MnNiCo-LDH-D outperforms MnNiCo-LDH-E in terms of specific capacitance. This is likely due to the more uniform distribution of Mn within the LDH layers in MnNiCo-LDH-D, enhancing the electroactive surface area and providing more accessible redox-active sites. These results reveal that optimizing transition metal ratios and doping levels is critical to balancing faradaic and capacitive contributions. This balance is essential for enabling high-rate capability in supercapacitor applications. The electrochemical behavior of the MnNiCo-LDH electrodes was further assessed using the GCD technique at various current densities. Figure S4a–e displays the GCD curves for MnNiCo-LDH-A, MnNiCo-LDH-B, MnNiCo-LDH-C, MnNiCo-LDH-D, and MnNiCo-LDH-E electrodes at current densities of 7.5, 10, 12.5, and 15 A/g, respectively. Notably, at lower current densities, specifically below 7.5 A/g, none of the electrodes could reach the full extent of the applied potential window during the charging phase. This incomplete charging likely stems from the internal resistance of electrode materials, which triggers redox reactions prematurely. This causes the onset of redox reactions before reaching the upper cutoff voltage, resulting in premature voltage plateaus. As expected, with an increase in current density, the discharge time of the electrodes decreases. At higher current densities, ion diffusion becomes restricted, thereby limiting the extent of faradaic reactions. The calculated specific capacitance values for each electrode at varying current densities were plotted to evaluate rate performance, as shown in Figure S4f. The capacitance retention values were found to be 91.95, 93.15, 88.37, 86.73, and 81.48% for MnNiCo-LDH-A, MnNiCo-LDH-B, MnNiCo-LDH-C, MnNiCo-LDH-D, and MnNiCo-LDH-E, respectively. Among these, MnNiCo-LDH-A and MnNiCo-LDH-B exhibited superior capacitance retention, suggesting their excellent rate capabilities and efficient charge transport dynamics. Interestingly, MnNiCo-LDH-B slightly outperformed MnNiCo-LDH-A, which may be attributed to its higher cobalt content. The presence of additional Co ions could enhance electrical conductivity and facilitate more reversible redox reactions, thereby improving charge–discharge efficiency at higher current densities. These findings emphasize the importance of compositional tuning in LDH-based materials. Tuning Ni, Co, and Mn ratios effectively controls internal resistance, redox kinetics, and rate performance, which are key to high-efficiency supercapacitor electrodes.

### 3.3 Full Cell Performance

To assess the practical applications of the trimetallic MnNiCo-LDH electrode, an asymmetric supercapacitor (ASC) was fabricated. A schematic representation of the MnNiCo-LDH-A//graphene-based ASC is presented in Figure 6a. Where, MnNiCo-LDH-A/NF serves as the cathode and graphene/NF as the anode in a 3 M KOH aqueous electrolyte. The electrochemical performance of the individual electrodes was first assessed by CV at a scan rate of 20 mV/s, as shown in Figure 6b. The graphene/NF electrode exhibited typical EDLC behavior, evidenced by a symmetrical CV curve within the −0.1 to −1.0 V potential window. In contrast, the MnNiCo-LDH-A/NF electrode demonstrated battery type electrochemical behavior within the potential range from −0.1 to 0.55 V. Further electrochemical analysis of the full cell was conducted to evaluate the viability of the voltage window. The CV curve of the asymmetric supercapacitor, displayed in Figure 6c, was obtained at the same scan rate of 20 mV/s. Notably, extending the potential window to 1.6 V introduced a weak oxygen evolution reaction peak, indicating the onset of parasitic side reactions. These side reactions are detrimental as they reduce the usable voltage window. Consequently, the maximum operational potential window of the asymmetric supercapacitor was determined to be 1.5 V, beyond which significant side reactions compromise performance. To explore the charge–discharge behavior further, GCD curves of the asymmetric supercapacitor were recorded at different potential windows, as shown in Figure 6d. The GCD curves revealed the presence of redox plateaus, indicative of the battery type nature of the MnNiCo-LDH electrode. During the charge/discharge process, a premature voltage plateau was observed at the upper limit of the potential window. This plateau is attributed to the onset of parasitic side reactions, particularly oxygen evolution, which suppresses further voltage increase and results in a self-limiting behavior of the system, especially in the alkaline electrolyte. The electrochemical performance of the ASC was also evaluated at varying scan rates and current densities. The CV curves recorded at different scan rates, presented in Figure 6e, show an increase in the integral area with increasing scan rate, although the redox peaks shifted slightly. This shift suggests that the electrochemical kinetics of the device are somewhat affected by the scan rate. Nevertheless, the overall shape of the CV curves remains consistent, reflecting good reversibility and stability of the device. The GCD curves demonstrated strong symmetry confirming the excellent electrochemical activity of the ASC (Figure 6f). Finally, to evaluate the energy and power densities of the ASC, a Ragone plot was constructed, as shown in Figure 7a. The plot illustrates the correlation between the energy and power densities, providing insight into the trade-off between energy storage and the ability to deliver power. To calculate the energy density (*E*) and power density (*P*), eq 8 and 9 were used as follows.^55^

$$E=\frac{C_{F}\times (\Delta V{)}^{2}}{2\times 3.6}$$ 8

$$P=\frac{E\times 3600}{\Delta t}$$ 9

where *C_F_* is the capacitance (F/g), *t* is the discharge duration (s), and Δ*V* is the potential window (V). As shown in the Ragone plot, the higher energy density of 36.9 Wh/kg was achieved at the power density of 473.4 W/kg. Even at the highest power density of 1960 W/kg, the energy density of ASC was maintained at around 19.1 Wh/kg. To ensure a fair evaluation, the performance of the fabricated MnNiCo-LDH//graphene ASC device is compared with previously reported trimetallic LDH-based asymmetric ASC devices. As depicted in the Ragone plot, the MnNiCo-LDH//graphene device exhibited superior energy and power densities compared to several previously reported materials. For instance, NiCoFe-LDH//activated carbon (AC) (8.7 Wh/kg@62.8 W/kg),^56^ Ni–Zn–Fe LDH//AC (14.9 Wh/kg@1077.6 W/kg),^57^ SWCNT/Ni–Co–Mn LDH//SWCNT (11.17 Wh/kg@66.2 W/kg),^58^ MnCo-LDH/NiCo-LDH//AC (36.9 Wh/kg@750 W/kg),^29^ NiCoMn-LDH/NG//AC (31.8 Wh/kg@473.4 W/kg),^59^ NiCo_2_Cr_1.5_–OH//Carbon (33.6 Wh/kg@374.6 W/kg),^60^ and Co–Fe LDH@NiO-Ni//AC (22 Wh/kg@800 W/kg).^61^ These results highlight the enhanced electrochemical performance of the MnNiCo-LDH//graphene device. This improvement can be attributed to the optimized composition of Ni, Co, and Mn in the LDH structure. The synergistic interaction among the three metal ions mitigates the adverse effects of phase transitions on layer spacing. This helps maintain structural stability during charge/discharge cycles. This phase transition can alter the ion diffusion paths and negatively impact the overall electrochemical performance. By stabilizing layer spacing, the flower-like MnNiCo-LDH ensures efficient ion transport, accelerating redox kinetics and overall electrochemical performance. Furthermore, the flower-like morphology of the MnNiCo-LDH contributes to its superior performance. The unique morphology offers a large surface area and abundant active sites, enhancing electrolyte interaction and improving energy storage capacity. The combined effects of structural optimization, metal ion synergy, and unique morphology endow the ASC device with enhanced performance, making it a promising candidate for energy storage applications. The stability of the ASC was tested in 3 M KOH at the current density of 2.5 A/g. In Figure 7b, the retention of *C*_F_ and Coulombic efficiency are shown as a function of the cycle number. It was found that 95.2% of the initial capacitance persisted after 10,000 GCD cycles. A Coulombic efficiency of around 96.6% was achieved during the whole process of charge and discharge. The improved stability of the MnNiCo-LDH can be attributed to the incorporation of Mn and its flower-like morphology. The addition of Mn enhances the material’s robustness, enabling it to better accommodate volume changes during cycling. Typically, MnCo-LDH demonstrates excellent stability performance, mitigating structural degradation and enhancing long-term cycling ability. In addition, the flower-like morphology plays a key role in improving the material’s resistance to volume expansion over extended use. As a result, the MnNiCo-LDH//graphene ASC achieves excellent electrochemical performance. Furthermore, the morphology of the MnNiCo-LDH was verified before and after the stability test, and the corresponding results are displayed in Figure 7c,d, respectively. The FESEM analysis revealed that the flower-like morphology was retained after repeated charge–discharge cycles. Nevertheless, the nanosheets anchored to the spherical structures remained well separated, with only slight compaction induced by electrolyte interactions. Figure S5 shows the XRD pattern of MnNiCo-LDH/NF before and after stability. The strong Ni foam peaks dominate the XRD patterns, masking the MnNiCo-LDH reflections before and after cycling. Notably, the overall XRD profiles remain unchanged after cycling, with no new peaks or significant shifts, suggesting the absence of phase transformation. This, together with the preserved morphology from FESEM analysis, supports the structural stability of the material during long-term cycling.

**Figure 6:**
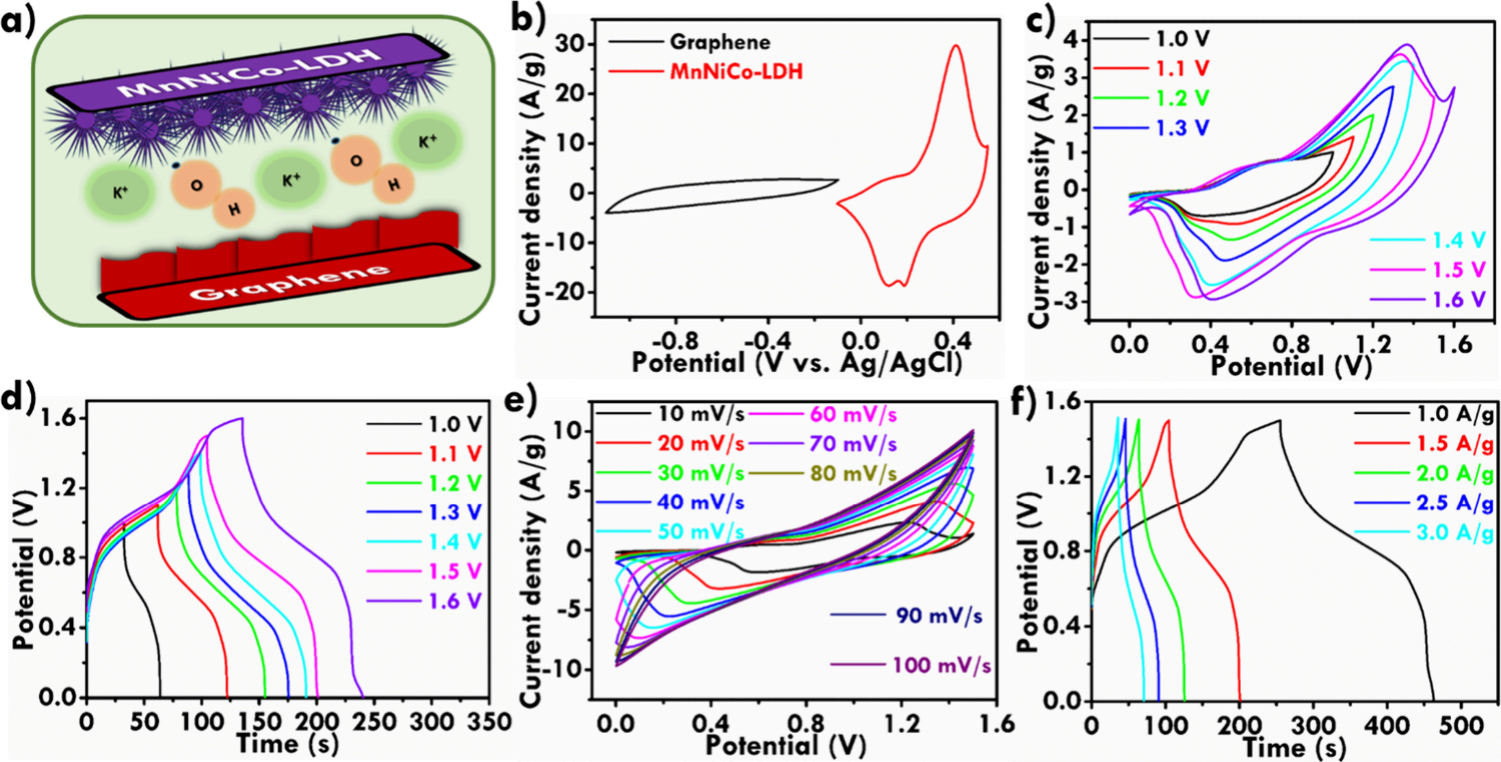
(a) Schematic diagram of the MnNiCo-LDH//graphene full cell design, (b) CV curves for the graphene and MnNiCo-LDH electrodes, (c) CV curves of the MnNiCo-LDH//graphene ASC full cell performance at different potential windows, (d) GCD curves of the MnNiCo-LDH//graphene ASC at various potential windows, (e) CV curves of ASC at different scan rates, and (f) GCD curves of ASC at various current densities.

**Figure 7:**
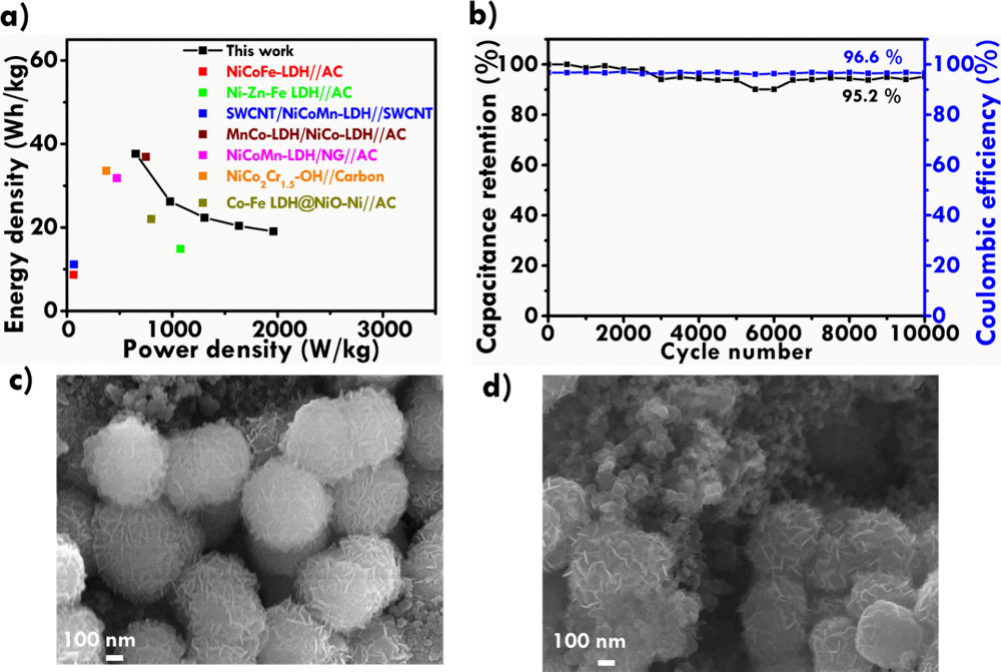
(a) Ragone plot, and (b) cycling stability of the ASC conducted at 2.5 A/g for 10,000 continuous GCD cycles; FESEM images of MnNiCo-LDH-A (c) before and (d) after stability test.

## 4 Conclusions

In this study, a flower-like trimetallic MnNiCo-LDH was successfully synthesized via a metal glycerolate-derived eco-friendly strategy. The incorporation of Mn and the synergistic interaction among Ni, Co, and Mn significantly enhanced the electrical conductivity and structural stability of the material. The synergistic balance of redox-active species, the well-ordered LDH structure, and the stable flower-like morphology collectively enhances the specific capacitance. Further, this synergy effectively suppressed phase transformation during repeated charge–discharge cycles, thereby improving long-term electrochemical durability. The fabricated MnNiCo-LDH//graphene ASC delivered a high energy density of 36.9 Wh/kg at a power density of 473.4 W/kg. The device also exhibits excellent cycling stability, retaining 95.2% of its initial capacitance after 10,000 cycles, confirming its reliability under practical operating conditions. These findings emphasize that rational compositional engineering combined with structural design is an effective strategy for optimizing LDH-based electrode materials.

## Supplementary Material



## Notes

Chemical reagents and characterization techniques; XRD results of NiCo-Glycerolate and MnNiCo-LDH-A; FTIR spectra of NiCo-Glycerolate and MnNiCo-LDH-A; XPS survey spectrum of MnNiCo-LDH-A; CV curves at different scan rates; GCD curves at different scan rates; and XRD results-after stability (PDF)
