# Supplementary material for: Self-Templated Metal Glycerolate-Derived Trimetallic Layered Double Hydroxides with Tunable Metal Cation Concentration for High-Performance Supercapacitors
Source: ACS Omega. 2026 May 16;11(21):31663–74. doi: 10.1021/acsomega.6c02482 (PMC13234675; doi:10.1021/acsomega.6c02482)
Supplement: Supplementary file 1 [file ao6c02482_si_001.pdf]

Supporting Information (SI)

Self-Templated Metal Glycerolate Derived Trimetallic Layered Double Hydroxides with Tunable Metal Cation Concentration for High-Performance Supercapacitors

Subbiramaniyan Kubendhiran[a], Nattha Arungwutthiwong[b], Thanapon Sripracha[b], Natkrit Kongphichphan[b], Hung-Ming Chen[c], Chutima Kongvarhodom[b*], Yung-Fu Wu[d*], Lu Yin Lin[a*]

[a]Department of Chemical Engineering and Biotechnology, National Taipei University of Technology, Taipei, 10608, Taiwan

[b]Department of Chemical Engineering, King Mongkut's University of Technology Thonburi, 126 Pracha-u-thit, Toong-kru, Bangkok 10140, Thailand

[c]Gingen technology Co., LTD., Rm. 7, 10F., No.189, Sec. 2, Keelung Rd., Xinyi Dist., Taipei 11054, Taiwan

[d]Department of Chemical Engineering, Ming Chi University of Technology, New Taipei City 24301, Taiwan

[*]Corresponding author (C. Kongvarhodom): E-mail: chutima.kon@kmutt.ac.th

[*]Corresponding author (Y.F. Wu): Email: gausswu@mail.mcut.edu.tw

[*]Corresponding author (L.Y. Lin): E-mail: lylin@ntut.edu.tw

Chemical reagents and characterization techniques

Nickel (II) nitrate hexahydrate ($Ni(NO_3)_2 \cdot 6H_2O$, Acros, 98%), cobalt (II) nitrate hexahydrate ($Co(NO_3)_2 \cdot 6H_2O$, Acros, 98%), isopropyl alcohol (Echo, 99.5%), glycerol (Echo, 99.5%), absolute ethanol (Honeywell, 99.8%), and manganese (II) nitrate tetrahydrate ($Mn(NO_3)_2 \cdot 4H_2O$, thermos scientific, 98%) reagentws were purchased and used without purification.

The structural morphology was analyzed using transmission electron microscopy (TEM) and field-emission scanning electron microscopy (FE-SEM, Nova NanoSEM 230, FEI, Oregon, USA). The crystalline property and elemental compositions of the materials were tested via X-ray diffraction (XRD, X'Pert3 Powder, PANalytical), energy-dispersive X-ray Spectroscopy (EDX), and X-ray photoelectron spectroscopy (XPS, JPS-9030, JEOL Ltd., Japan). The electrochemical characteristics of MnNiCo-LDH samples were assessed using a three-electrode system. The working electrodes were prepared by mixing the active material, acetylene black, and polyvinylidene fluoride (PVDF) in a weight ratio of 70:20:10 using 0.5 mL of 1-methyl-2-pyrrolidone (NMP) as the solvent. The mixture was homogenized by ball milling for 3 min, repeated seven times to ensure uniform dispersion. The resulting slurry was then coated onto a nickel foam (1 cm^2) substrate. Finally, the electrodes were dried at 60 °C for 10 h in air. Cyclic voltammetry (CV) and galvanostatic charge-discharge (GCD) tests were performed using the FRA2 module (PGSTAT 204, Autolab, Eco–Chemie, the Netherlands). Electrochemical impedance spectroscopy (EIS) was tested out from 100 kHz to 0.01 Hz. In the three-electrode configuration, the fabricated MnNiCo-LDH electrode served as the working electrode, Ag/AgCl as the reference electrode, and a platinum wire as the counter electrode, using 3 M potassium hydroxide (KOH, Showa chemicals, 85%) as the electrolyte.

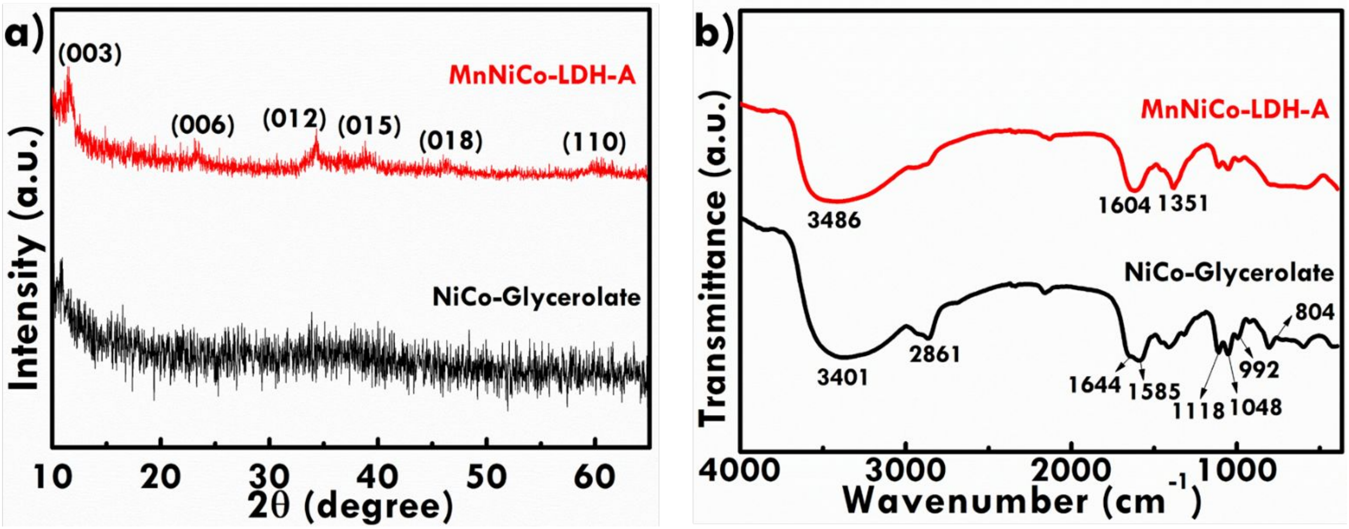

Fig. S1 a) XRD and b) FTIR spectra of NiCo-glycerolate and MnNiCo-LDH-A.

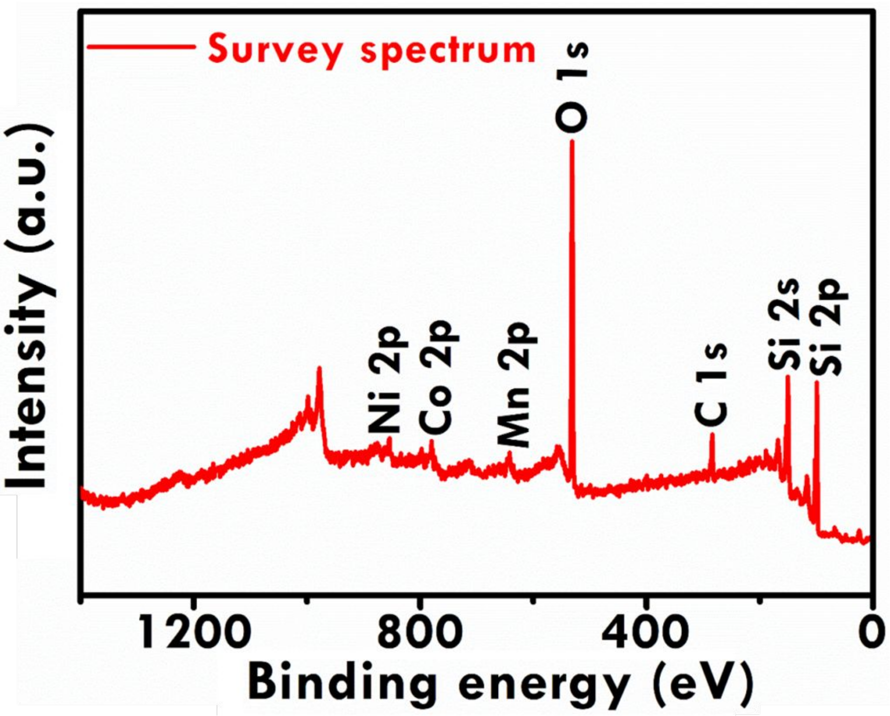

Fig. S2 XPS survey spectrum of MnNiCo-LDH-A.

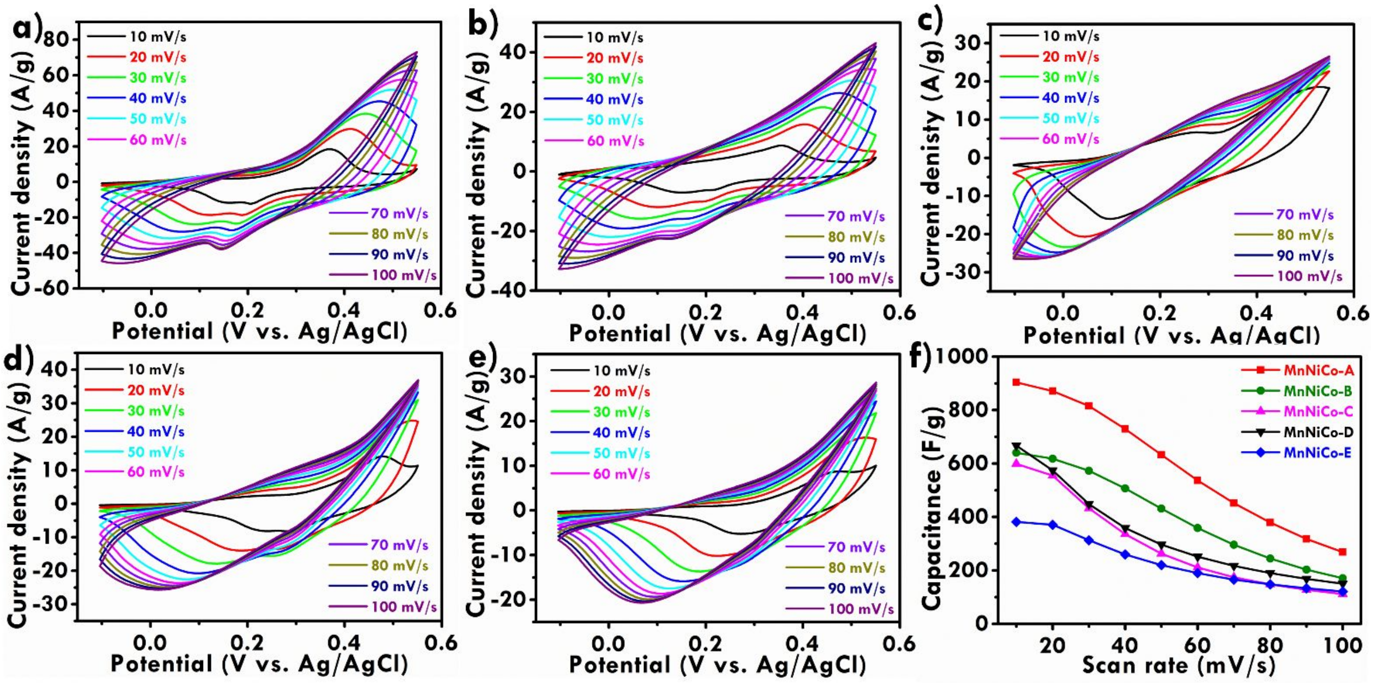

Fig. S3 CV curves recorded at different scan rates from 10 to 100 mV/s for (a) MnNiCo-LDH-A, (b) MnNiCo-LDH-B, (c) MnNiCo-LDH-C, (d) MnNiCo-LDH-D, and (e) MnNiCo-LDH-E electrodes; (f) relationships of the capacitance and scan rates.

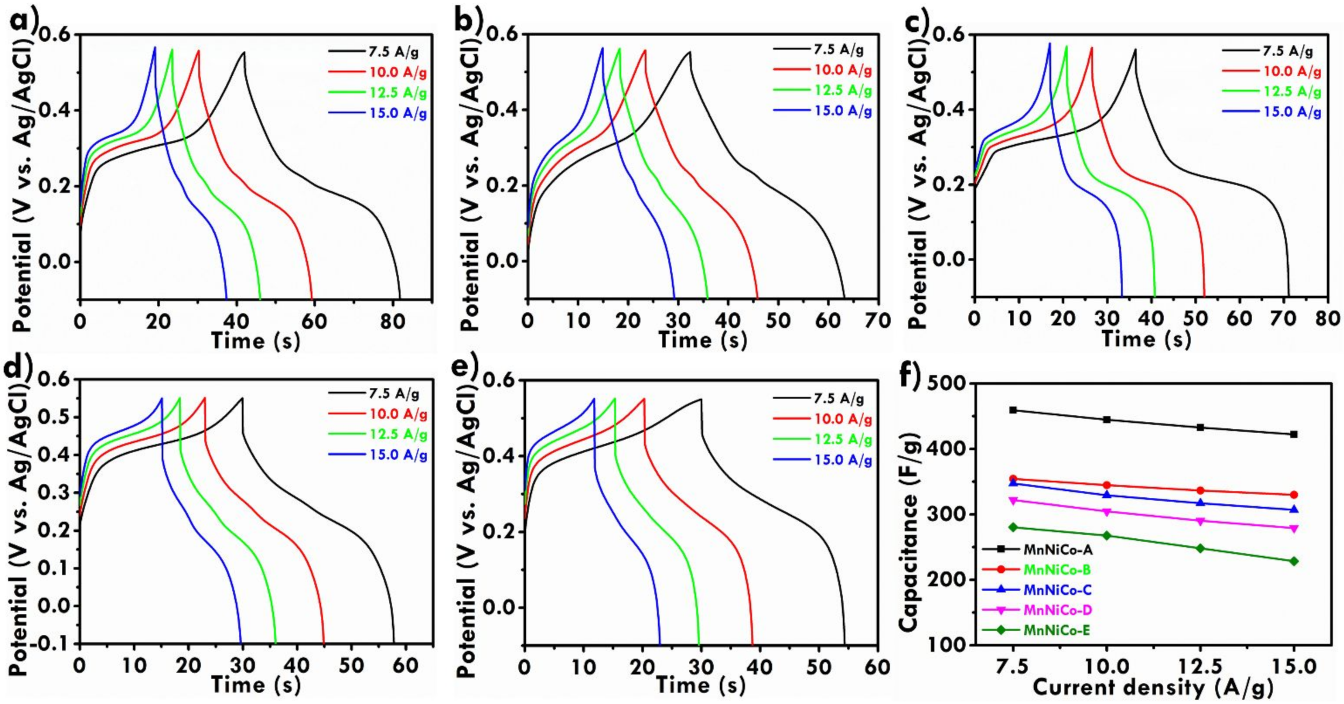

Fig. S4 GCD curves recorded at current densities from 7.5 to 15.0 A/g for (a) MnNiCo-LDH-A, (b) MnNiCo-LDH-B, (c) MnNiCo-LDH-C, (d) MnNiCo-LDH-D, and (e) MnNiCo-LDH-E electrodes; (f) relationships of the capacitance and current densities.

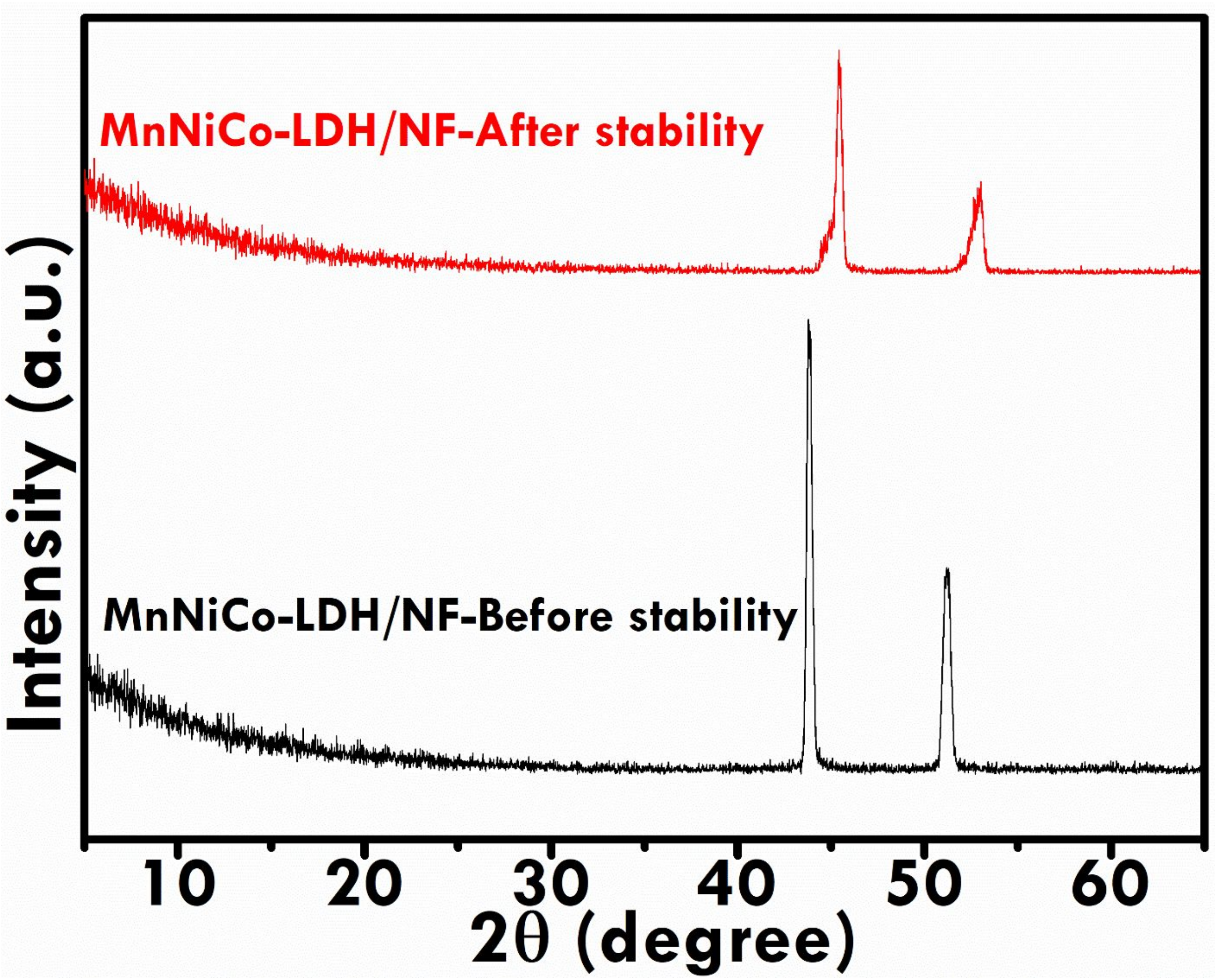

Fig. S5 XRD pattern of MnNiCo-LDH/NF before and after stability.
